# Patterns of comorbidity in community-dwelling older people hospitalised for fall-related injury: A cluster analysis

**DOI:** 10.1186/1471-2318-11-45

**Published:** 2011-08-18

**Authors:** Trang Vu, Caroline F Finch, Lesley Day

**Affiliations:** 1 Monash Injury Research Institute, Monash University, Victoria 3800, Australia

**Keywords:** comorbidity, patterns, cluster analysis, elderly, falls prevention

## Abstract

**Background:**

Community-dwelling older people aged 65+ years sustain falls frequently; these can result in physical injuries necessitating medical attention including emergency department care and hospitalisation. Certain health conditions and impairments have been shown to contribute independently to the risk of falling or experiencing a fall injury, suggesting that individuals with these conditions or impairments should be the focus of falls prevention. Since older people commonly have multiple conditions/impairments, knowledge about which conditions/impairments coexist in at-risk individuals would be valuable in the implementation of a targeted prevention approach. The objective of this study was therefore to examine the prevalence and patterns of comorbidity in this population group.

**Methods:**

We analysed hospitalisation data from Victoria, Australia's second most populous state, to estimate the prevalence of comorbidity in patients hospitalised at least once between 2005-6 and 2007-8 for treatment of acute fall-related injuries. In patients with two or more comorbid conditions (multicomorbidity) we used an agglomerative hierarchical clustering method to cluster comorbidity variables and identify constellations of conditions.

**Results:**

More than one in four patients had at least one comorbid condition and among patients with comorbidity one in three had multicomorbidity (range 2-7). The prevalence of comorbidity varied by gender, age group, ethnicity and injury type; it was also associated with a significant increase in the average cumulative length of stay per patient. The cluster analysis identified five distinct, biologically plausible clusters of comorbidity: cardiopulmonary/metabolic, neurological, sensory, stroke and cancer. The cardiopulmonary/metabolic cluster was the largest cluster among the clusters identified.

**Conclusions:**

The consequences of comorbidity clustering in terms of falls and/or injury outcomes of hospitalised patients should be investigated by future studies. Our findings have particular relevance for falls prevention strategies, clinical practice and planning of follow-up services for these patients.

## Background

Community-dwelling older people aged 65+ years sustain falls frequently--28% - 35% fall at least once annually [1-7], while 9%-14% experience multiple falls each year [5-7]. The highest proportions of community-dwelling older people who fall are in the 80+ years age group [2,7]. Nearly half to 60% of all falls result in physical injuries [7-11], and 20%-50% of these require medical attention including emergency department (ED) care and/or hospitalisation [1,8,12]. In 2006, 10% ED visits by older people in the United States (US) was for injurious fall. Those seen in ED and subsequently admitted were more than twice as likely to be discharged to long-term care facilities than ED patients admitted for other conditions [13].

A recent systematic review of observational studies on risk factors for falling in community-dwelling older people shows that certain health conditions and impairments contribute independently to the risk of falling or experiencing a fall injury [14]. This suggests that individuals with these conditions or impairments should be the focus of falls prevention provided that effective interventions are available. Since older people commonly have multiple conditions/impairments [15], knowledge about which conditions/impairments coexist and contribute to an increased risk of falls/fall injury would be valuable in the targeting of appropriate interventions. The prevalence of coexisting conditions and impairments (hereafter referred to as comorbidity) in community-dwelling older fallers has been investigated in a limited number of studies [3,9,16]. However, to date, studies investigating the clustering patterns of comorbidity in patients with fall-related injury are lacking. The objective of our study was to describe the epidemiology of hospitalised, acute fall-related injuries in community-dwelling older people aged 65+ years, and in particular examine the prevalence and patterns of comorbidity in this population group.

## Methods

We analysed the Victorian Admitted Episodes Dataset (VAED) for three successive fiscal years 2005-6, 2006-7 and 2007-8. The VAED is an administrative data collection of admitted patient episodes in hospitals in the state of Victoria, Australia's second most populous state. It is managed by the Victorian Department of Health (DOH) and used to support casemix funding, epidemiological research, health services planning and policy development [17]. The collection is subject to regular audits which indicate good-to-excellent diagnosis and procedure coding quality [18]. Administrative, demographic and clinical information is collected for each episode of care. Each patient within a hospital is identified by a unique, hospital generated patient identifier and each episode has a unique hospital derived episode number; however, the VAED lacks a system-wide unique patient identifier [17]. Episodes containing an external cause of injury in the range of W00-W19 in the International Classification of Diseases, Tenth Revision, Australian Modification (ICD-10-AM, 4th or 5th editions) [19,20] were extracted from the VAED and internally linked by the DOH using all available identifiers and step wise deterministic linkage to produce a linked dataset for the present study (L Sundaresan, personal communication 2009). The final dataset contained a linkage derived patient identification number, but no personally identifiable information.

Patients were included in our study if they had at least one incident fall-related injury admission, defined as a hospital admission with a principal diagnosis in the range of S00 to T75 or T79 in ICD-10-AM and a source coded as "private residence/accommodation" [19-21]. In order to accurately identify patients with incident hip fractures we selected only emergency hospital admissions for acute care with no hip revision procedure code(s) [22]. We calculated a patient's cumulative length of stay in hospital for 2005-6 to 2007-8 from the first incident injury admission. Patients' socioeconomic status (SES) was determined by linking their postcode of residence with an index of economic and social resources of households measured at postcode level [23].

We assessed the prevalence of comorbidity among study patients at the time of their first incident fall-related injury admission using the Deyo adaptation of the Charlson Comorbidity Index (CCI) because this index was constructed using administrative data similar to those collected for the VAED, and validated using the VAED [24]. We also estimated the prevalence of other risk factors for falls and fractures, including osteoporosis, Parkinson's disease, ataxia, visual impairment, deafness and delirium, using ICD-10-AM codes also tested on the VAED [25]. We distinguished comorbidities from adverse events that arose during hospitalisation by utilising a condition-onset flag available in the VAED [17]. For patients with more than one hospitalisation for the first incident injury, we optimised comorbidity ascertainment by defining the first multiday record as the index hospitalisation and searching this record as well as looking back at previous record(s) for the presence of comorbidities (hereafter referred to as lookback) [26]. Comorbidity was deemed to be present if it was coded in one or more of these records. The first multiday record was used because the number of codes for comorbid conditions was significantly less in same-day and overnight-stay records than in multiday records.

**Figure 1:**
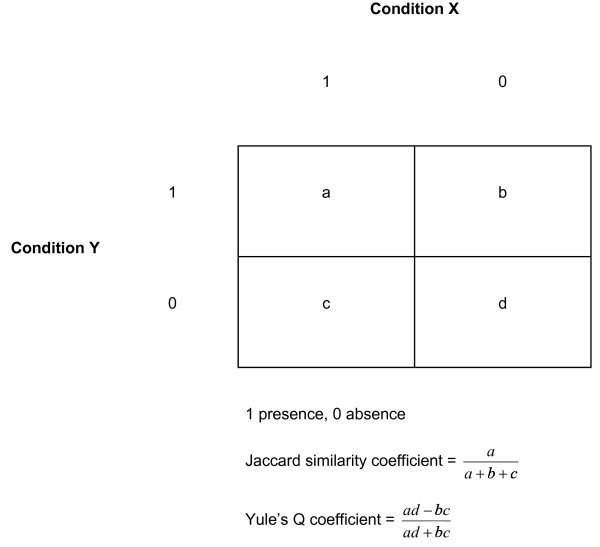
**Two-by-two contingency table for binary comorbidity data**.

In patients with two or more comorbid conditions (multicomorbidity) we used an agglomerative hierarchical clustering method to cluster comorbidity variables and identify constellations of conditions [27]. These variables took the value of one when a given comorbidity was present and zero when it was absent. Under this method, each comorbidity began as an individual cluster which was gradually merged with the most similar other clusters until a single cluster containing all comorbidities was obtained. We chose the agglomerative approach because we did not know the possible number of clusters a priori. The hierarchical strategy was followed based on published research which suggests that certain co-occurring diseases tend to have common underlying risk factors or genetic predisposition [28]. We chose the average linkage method to measure the distance between two clusters because this method is considered to be suitable for most clustering situations and sufficiently robust [29]. We used the Jaccard binary similarity coefficient, representing measures of co-occurrence and judged as a reasonable choice for most applications involving binary data, to assess the similarity between pairs of comorbidities (see Figure 1) [29]. To test the robustness of this choice we used Yule's Q coefficient, which represents measures of association (Figure 1), in a sensitivity analysis. We also tested the robustness of the clustering solutions by conducting cluster analysis on subgroups of patients defined by gender, ethnicity, age group and injury type. Both the Calinski/Harabasz index [30] and the Duda/Hart index [31] were used to help determine the number of clusters. Finally, the literature on comorbidity was used to assist with the interpretation of the generated cluster trees (dendrograms). We excluded comorbidities with very low prevalence (< 2.0%) from the cluster analysis to minimise chaining (sequential joining of low prevalent comorbidities into existing clusters) [29].

The Monash University Human Research Ethics Committee granted approval for this study (ID CF09/0759 - 2009000332). We conducted all analyses in Stata version 11 [32]. We evaluated equality of proportions using two-sample chi square tests of proportions, or Fisher's exact test, as appropriate. For skewed continuous variables, we compared medians using nonparametric K-sample tests on the equality of medians [32]. The existence of a trend over time in hospitalised fall-related injuries was tested using a chi-square test for trend. The association between cluster membership and demographic and clinical variables was explored using a multivariate logistic regression model. All tests were two tailed with a level of significance of 5%.

## Results

Approximately 45 thousand community-dwelling older people aged 65+ years were hospitalised at least once between 2005-6 and 2007-8 for treatment of acute fall-related injuries (Table 1). The median age was 82 years; 80 years for men and 82 years for women. Women outnumbered men in each year; however, the proportions of women hospitalised for acute fall-related injuries declined significantly over time (χ^2 ^for trend 23.3, p < 0.001). The majority of hospitalised fallers were non-Indigenous and born in English-speaking countries (ESC). We observed a statistically significant downward trend in the proportion of fallers born in ESC accompanied by a statistically significant upward trend in the proportion of fallers born in non-English-speaking countries (NESC) (χ^2 ^for trend 27.1, p < 0.001). The three most frequently recorded NESC were Italy (24.1%), Greece (11.8%) and Germany (6.3%).

**Table 1:** Patients' characteristics at admission.

	2005-6 n = 15,244	2006-7 n = 15,109	2007-8 n = 14,589	Total N = 44,942
Median (IQ) age in years	82 (76-87)	82 (76-87)	82 (75-87)	82 (76-87)
Women	10,893 (71.5)	10,627 (70.3)	10,052 (68.9)	31,572 (70.3)
Married	5,870 (38.5)	5,992 (39.7)	6,116 (41.9)	17,978 (40.0)
Non-Indigenous	15,209 (99.8)	15,040 (99.5)	14,477 (99.2)	44,726 (99.5)
Hospital insurance	4,611 (30.3)	4,683 (31.0)	4,549 (31.2)	13,843 (30.8)
ESC*	11,721 (76.9)	11,403 (75.5)	10,838 (74.3)	33,962 (75.6)
Most disadvantaged†	1,931 (12.9)	2,018 (13.6)	1,953 (13.6)	5,902 (13.3)
Least disadvantaged‡	2,076 (13.8)	1,984 (13.3)	1,880 (13.1)	5,940 (13.4)

Values are numbers (percentages) unless stated otherwise

IQ = Interquartile range. ESC = English-speaking country of birth.

* Nine patients across the three fiscal years had missing country of birth.

† The lowest decile of the socio-economic index.

‡ The highest decile of the socio-economic index.

Almost 60% of first incident fall-related injury admissions in community-dwelling older people aged 65+ were for fractures (Table 2). Three quarters of the patients with fractures were women. Women were also over-represented in other types of injuries (61.9%-68.0%). Fracture sites included hip (33.9%), wrist (12.6%), shoulder and upper arm (12.0%), lower leg including ankle (9.2%), pelvic (7.7%), and skull and facial bones (2.6%).

**Table 2:** Nature of patients' first incident injury.

Nature of injury	2005-6 n = 15,244	2006-7 n = 15,109	2007-8 n = 14,589	Total N = 44,942
Fractures	9,109 (59.8)	8,922 (59.1)	8,474 (58.1)	26,505 (59.0)
Open wounds	1,767 (11.6)	1,706 (11.3)	1,601 (11.0)	5,074 (11.3)
Superficial injuries	930 (6.1)	963 (6.4)	926 (6.4)	2,819 (6.3)
Dislocation, sprain & strain	566 (3.7)	540 (3.6)	580 (4.0)	1,686 (3.8)
Other*	2,872 (18.8)	2,978 (19.7)	3,008 (20.6)	8,858 (19.7)

Values are numbers (percentages) unless stated otherwise

* Miscellaneous injuries including injury to muscle and tendons, intracranial injury, traumatic amputation, injury to internal organs, injury to blood vessels, injury to nerves and spinal cord, and eye injury.

Nearly one-third of first incident injury admissions were due to a fall on the same level from tripping or slipping (Table 3). Another one-fifth of the admissions were the result of other falls on the same level. The home was the most common place of occurrence of the falls which gave rise to these admissions. However, the activity at the time of a fall was only recorded in about a third of the admissions.

**Table 3:** Circumstances of patients' first incident injury.

Mechanism of falls	2005-6 n = 15,244	2006-7 n = 15,109	2007-8 n = 14,589	Total N = 44,942
Fall on same level from tripping or slipping	4,696 (30.8)	4,547 (30.1)	4,478 (30.7)	13,721 (30.5)
Other fall on same level	3,079 (20.2)	3,125 (20.7)	2,951 (20.2)	9,155 (20.4)
Other mechanisms	2,970 (19.5)	3,015 (20.0)	2,946 (20.2)	8,931 (19.9)
Unspecified fall mechanism	4,499 (29.5)	4,422 (29.3)	4,214 (28.9)	13,135 (29.2)
**Location of falls**				
Home^†^	7,281 (47.8)	7,214 (47.8)	7,033 (48.2)	21,528 (47.9)
Café/restaurant, shop, sidewalk and roadway	1,152 (7.6)	1,230 (8.1)	1,182 (8.1)	3,564 (7.9)
Other specified place of occurrence	4,060 (26.6)	3,921 (26.0)	3,672 (25.2)	11,653 (25.9)
Unspecified place of occurrence	2,751 (18.1)	2,744 (18.2)	2,702 (18.5)	8,197 (18.2)
**Activity at time of the fall**				
While engaging in vital activities^§^	1,813 (11.9)	1,784 (11.8)	1,751 (12.0)	5,348 (11.9)
Sports and exercise activity	122 (0.8)	119 (0.8)	102 (0.7)	343 (0.8)
Other	3,317 (21.8)	3,259 (21.6)	3,311 (22.7)	9,887 (22.0)
Unspecified	9,992 (65.6)	9,947 (65.8)	9,425 (64.6)	29,364 (65.3)

Values are numbers (percentages) unless stated otherwise

† Including driveway to home.

§ Resting, sleeping eating or engaging in other vital activities.

Table 4 presents estimates of the comorbidity prevalence among patients hospitalised for treatment of acute fall-related injuries stratified by gender. More than one in four patients had at least one comorbid condition and among patients with comorbidity one in three had multicomorbidity (range 2-7). The average cumulative length of stay (LOS) per patient for 2005-6 to 2007-8 was 26.8 days (95% confidence interval 26.3 to 27.4) for patients with comorbidity and 17.1 days (16.8 to 17.4) for patients without comorbidity. Of the patients with comorbidity, those with multicomorbidity had higher average cumulative LOS for 2005-6 to 2007-8 (28.8 days, 27.9 to 29.8) than those with just one condition (25.9 days, 25.3 to 26.6).

**Table 4:** Prevalence of comorbidity by gender.

	Men n = 13,370	Women n = 31,572	Total N = 44,942
Median (IQ) age adjusted CCI^†^	5 (4-6)	5 (4-6)	5 (4-6)
Any comorbid condition	30.7 (30.0-31.5)	23.7 (23.2-24.2)	25.8 (25.4-26.2)
Diabetes^‡^	10.0 (9.5-10.6)	7.0 (6.7-7.3)	7.9 (7.6-8.1)
Renal disease	6.8 (6.3-7.2)	3.9 (3.7-4.1)	4.8 (4.6-5.0)
Dementia	3.7 (3.4-4.1)	3.4 (3.2-3.6)	3.5 (3.3-3.7)
Congestive heart failure	3.4 (3.1-3.7)	2.7 (2.5-2.9)	2.9 (2.7-3.1)
Pulmonary disease	3.4 (3.1-3.7)	2.0 (1.8-2.1)	2.4 (2.2-2.5)
Parkinson's disease	3.1 (2.8-3.4)	1.4 (1.3-1.6)	1.9 (1.8-2.0)
Cerebral vascular accident	2.7 (2.4-3.0)	1.4 (1.2-1.5)	1.8 (1.6-1.9)
Cancer	2.6 (2.3-2.9)	1.0 (0.9-1.2)	1.5 (1.4-1.6)
Paraplegia	1.9 (1.7-2.1)	0.9 (0.8-1.0)	1.2 (1.1-1.3)
Delirium	1.5 (1.3-1.7)	1.2 (1.1-1.4)	1.3 (1.2-1.4)
Osteoporosis	1.2 (1.0-1.4)	3.3 (3.1-3.5)	2.7 (2.5-2.8)
Metastatic cancer	1.2 (1.0-1.4)	0.5 (0.4-0.6)	0.7 (0.6-0.8)
Acute myocardial infarction	1.1 (0.9-1.3)	0.9 (0.8-1.0)	0.9 (0.9-1.0)
Vision impairment	1.0 (0.8-1.2)	1.0 (0.9-1.1)	1.0 (0.9-1.1)
Deafness	0.8 (0.7-1.0)	0.6 (0.5-0.7)	0.7 (0.6-0.8)
Peripheral vascular disease	0.6 (0.5-0.8)	0.3 (0.3-0.4)	0.4 (0.4-0.5)
Liver disease	0.2 (0.1-0.3)	0.1 (0.0-0.1)	0.1 (0.1-0.1)
Ataxia	0.2 (0.1-0.3)	0.1 (0.1-0.2)	0.1 (0.1-0.2)
Connective tissue disorder	0.1 (0.1-0.2)	0.4 (0.3-0.4)	0.3 (0.3-0.4)
Severe liver disease	0.1 (0.1-0.2)	0.0 (0.0-0.1)	0.1 (0.0-0.1)
Peptic ulcer	0.1 (0.1-0.2)	0.1 (0.1-0.2)	0.1 (0.1-0.2)

Values are percentages (95% confidence intervals) unless stated otherwise*

IQ - interquartile range. CCI-Charlson comorbidity index.

* Neuromyalgia and HIV not shown because of very low prevalence (0.01% for each condition).

† Non-parametric K sample test for equality of medians.

‡ Diabetes with or without complications. The prevalence of diabetes with complications was 4.8% (4.5-5.2) in men, 3.3% (3.1-3.5) in women, and 3.7% (3.6-3.9) overall. The prevalence of diabetes without complications was 6.1% (5.7-6.5) in men, 4.0% (3.8-4.3) in women, and 4.7% (4.5-4.9) overall.

The overall prevalence of comorbidity was significantly higher in men than in women. Multicomorbidity was also significantly more prevalent in men than in women (11.7%, 11.2% to 12.3% vs. 6.8%, 6.6% to 7.1%). The prevalence of most individual comorbid conditions was also statistically significantly higher in men, except for acute myocardial infarction (AMI), dementia, liver disease, severe liver disease, peptic ulcer disease, ataxia, delirium, deafness and visual impairment. Women had a higher prevalence of osteoporosis and connective tissue disease than men. The top five comorbidities in men were diabetes (with or without complications), renal disease, dementia, congestive heart failure (CHF) and pulmonary disease (PUL). The five most common comorbidities in women differed slightly from those found in men--osteoporosis was the fourth most common condition while CHF ranked fifth and PUL was not included in the top five. The average cumulative LOS per patient for 2005-6 to 2007-8 was 27.9 days (27.2 to 28.6) for women with comorbidity and 24.9 days (24.0 to 25.8) for men with comorbidity.

In addition to gender differences, we also found differences in comorbidity according to injury types. Fracture patients had a higher prevalence of any comorbidity (27.4%, 26.9% to 28.0%) than patients with open wounds (20.2%, 19.1% to 21.3%), superficial injuries (23.9%, 22.4% to 25.6%), or dislocation, sprain and strain (14.9%, 13.2% to 16.7%); but not patients with all other types of injury (26.8%, 25.1% to 27.7%). Fracture patients also had a higher prevalence of osteoporosis (3.7%, 3.5% to 3.9%) than patients with open wounds (0.4%, 0.2% to 0.6%), superficial injuries (0.9%, 0.6% to 1.3%), dislocation, sprain and strain (1.0%, 0.6% to 1.6%) or other types of injury (1.7%, 1.4% to 2.0%).

The prevalence of comorbidity also varied significantly by ethnicity. Patients born in NESC were significantly more likely to have any comorbidity than those born in ESC (28.0%, 27.1% to 28.9% vs. 25.2%, 24.8% to 25.7%). Among patients with a comorbidity burden, 35.4% (33.6% to 37.2%) of those born in NESC had multicomorbidity compared with 31.3% (30.3% to 32.3%) in those born in ESC. Twelve percent (11.4% to 12.7%) of patients born in NESC had diabetes, compared with 6.7% (6.5% to 7.0%) of patients born in ESC. The prevalence of renal disease was also higher in patients born in NESC (6.5%, 6.0% to 6.9% vs. 4.3%, 4.1% to 4.5%). However, patients born in ESC had a higher prevalence of PUL (2.6%, 2.5% to 2.8% vs. 1.7%, 1.4% to 1.9%).

Variations in comorbidity prevalence were also observed with age. The prevalence of comorbidity was significantly higher among patients aged 75-84 years (the "old-old") (27.3%, 26.7% to 28.0%) and patients aged 85+ years (the "oldest-old") (27.9%, 27.2% to 28.6%), as compared to patients aged 65-74 years (the "young-old") (19.2%, 18.4% to 20.0%). Diabetes, renal diseases and osteoporosis were among the top five comorbidities in all age groups. Dementia was among the top five in the "old-old" and the "oldest-old" but not the "young-old". Similarly, CHF ranked fourth and sixth in the "oldest-old" and the "old-old", respectively, but ranked tenth in the "young-old".

In terms of SES, patients living in the most disadvantaged areas did not have a significantly higher prevalence of comorbidity than patients living in the least disadvantaged areas (27.5% (26.3% to 28.6%) vs. 26.6% (25.5% to 27.8%), respectively). The top five comorbidities in the former were the same as those for men while the top five comorbidities in the latter were the same as those for women, reflecting a higher proportion of men in the former than the latter (p < 0.05). About 6% (5.5% to 6.7%) of patients from the least disadvantaged areas had diabetes, compared with 9.5% (8.7% to 10.2%) from the most disadvantaged areas. Approximately 5% (4.1% to 5.2%) of patients in the former had dementia, compared with 3.1% (2.7% to 3.6%) in the latter. When direct standardisation of dementia prevalence rates was performed, the least disadvantaged group had a 44% (2005-6) to 82% (2006-7) higher dementia prevalence than the most disadvantaged group. No difference in dementia prevalence between these two SES groups was found for 2007-8.

**Figure 2:**
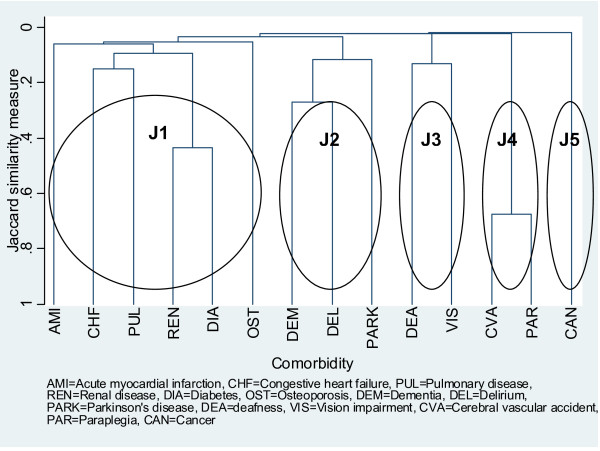
**Dendrogram based on comorbidity pattern of 3,729 patients constructed using average linkage and Jaccard coefficient**.

Figure 2 presents a dendrogram constructed using average linkage and Jaccard coefficients depicting the relationships between comorbidities in patients with multicomorbidity (n = 3,729). The vertical axis represents the Jaccard similarity coefficient at which clusters were joined, with higher values indicating more highly similar clusters. The dendrogram depicted five clusters--J1 representing cardiopulmonary/metabolic disorders and their sequelae, and containing osteoporosis, AMI, CHF, PUL, renal disease and diabetes; J2 representing neurological diseases and containing Parkinson's disease, delirium and dementia; J3 representing sensory conditions and containing deafness and visual impairment; J4 representing cerebral vascular accident (stroke) and its sequelae, and containing stroke and paraplegia; and J5 representing cancer.

Among the patients with multicomorbidity 53.1% had either diabetes or renal disease and 46.9% had neither suggesting that the J1 cluster was the largest cluster among the five clusters identified. An exploratory multivariate logistic regression model showed that several variables associated with the first incident injury admission, namely age, country of birth, region of residence, SES, patient account type (public, private or Department of Veterans' Affairs), discharge to nursing home, and intensive care unit admission, were significant predictors of J1 membership. For every year increase in age the odds of being in the J1 cluster increased by 2.3% (0.7% to 3.9%). Compared to patients born in ESC, those born in Europe, the former Union of Soviet Socialist Republics, and South and Central America had a 66.7% higher odds of being in the J1 cluster (39.4% to 99.1%) whereas patients born in Asia, the Middle East, Africa and Pacific islands had a 167.1% higher odds (88.5% to 278.4%). Being obese increased the odds for J1 membership more than 5-fold (237.4% to 1045.9%). Living in areas in the bottom two deciles of the SES index increased the odds for J1 membership between 35.4% and 50.9%. J1 membership also varied by geographical locations. The model was a good fit (p = 0.7 in the Hosmer-Lemeshow goodness-of-fit test) but was not highly predictive (area under the receiver operating characteristic curve was 0.6).

Figure 2 was identical or almost identical to corresponding dendrograms constructed for subgroups of patients with multicomorbidity defined by gender, ethnicity, presence of fracture and age group. Variations to the relationships between comorbidities were observed in the J1 cluster and involved the dendrograms for female patients (n = 2,160), male patients (n = 1,569), patients born in NESC (n = 973), the "young-old" patients (n = 581) and the "oldest-old" (n = 1,426) (dendrograms not shown). In Figure 2 for all patients, osteoporosis was the last comorbidity to join the J1 cluster. However, in the dendrogram for the "young-old", osteoporosis was linked directly with cardiopulmonary conditions. In the dendrogram for female patients and that for patients born in NESC, osteoporosis was linked directly with cardiopulmonary conditions, and diabetes and its sequelae. In contrast, in male patients and the "oldest-old", osteoporosis was grouped with sensory conditions. Additionally, in the former cancer was the last condition to join the J1 cluster.

**Figure 3:**
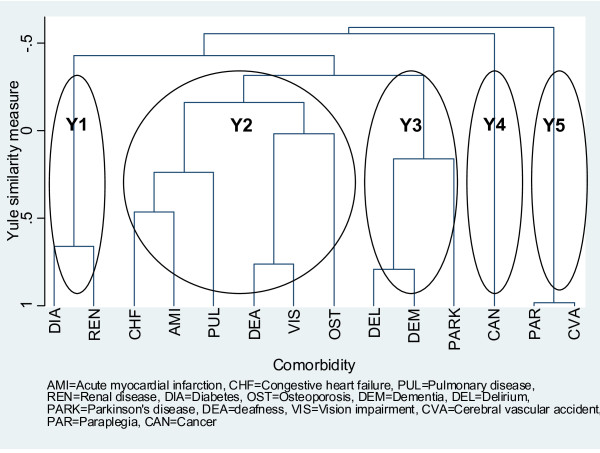
**Dendrogram based on comorbidity pattern of 3,729 patients constructed using average linkage and Yule's Q coefficient**.

Figure 3 presents a dendrogram for all patients with multicomorbidity based on Yule's Q coefficient (n = 3,729). The vertical axis denotes the Yule's Q similarity coefficient which ranges from minus one to one. Although this dendrogram also suggested five distinct clusters, two of these clusters differ from those in Figure 2 indicating that the choice of similarity coefficient did affect the resulting clustering solution. Three clusters, Y3, Y4 and Y5, were identical to clusters J2, J5 and J4 respectively. The remaining clusters were: Y1 representing diabetes and its sequelae, and containing diabetes and renal disease; and Y2 representing cardiopulmonary diseases and age-related conditions, and containing CHF, AMI, PUL, deafness, visual impairment and osteoporosis. Corresponding dendrograms obtained in the subgroup analyses were identical or almost identical to that presented in Figure 3, with the exceptions of three dendrograms--the dendrogram for female patients in which osteoporosis clustered with PUL and the resulting subcluster linked with remaining comorbidities within the Y2 cluster; the dendrogram for the "young-old" in which visual impairment joined with the Y1 cluster, deafness connected with dementia within the Y3 cluster and the relationship of osteoporosis with remaining comorbidities in the Y2 cluster was identical to that for female patients; and the dendrogram for the "old-old" (n = 1,722) in which sensory conditions clustered with cancer and osteoporosis was the last condition to connect within the Y2 cluster.

## Discussion

To our knowledge, this is the first population-based study to concurrently estimate the prevalence of comorbidity and describe the relationships between comorbidities in community-dwelling older people aged 65+ years hospitalised for fall-related injuries. Recently published, large comparable studies have omitted comorbidity from consideration [33,34] while less recent, smaller comparable studies only included a cursory assessment of comorbidity in which the range of comorbidities considered was limited and the patterns of comorbidity were not investigated [35]. Less comparable studies on injury hospitalisation which examined comorbidity in greater detail have largely focused on the impact of comorbidity on mortality and disregarded the patterns and clustering of comorbidity [36-38].

The median ages for men and women in our study are identical to those reported by a recent large US study [39]. Agreement between ours and previous studies regarding the proportion of main types of injury is also very good [33,39,40]. The proportion of patients born in ESC in our study reflects the pattern of ethnicity more generally in hospitalised patients aged 18+ years in Victoria, Australia [25]. The proportion of women in our study (70.3%) is slightly lower than that reported by previous Australian and international studies (71.2%-74.3%) [33,34]. However, these studies included nursing home residents which would increase the proportion of women due to the overrepresentation of women in nursing homes [41].

The overall prevalence of comorbidity in our study population (25.8%) is within the range of estimates for hospitalised patients aged 18+ years in Victoria (24.1%-30.6%) [24]. However, our estimate is low compared to US Medicare elderly injured patients (45.3%) [42] but is higher than Italian elderly injured patients (18.0%) [43]; both of which contained injuries due to falls. These differences may be explained by the inclusion of nursing home residents in the former study, and the provision of a more restricted set of diagnoses in the latter study. Our estimate of the prevalence of diabetes is in excellent agreement with that reported in another US study [35]. Our finding of the differential prevalence of diabetes among SES groups is also consistent with the literature [44]. The prevalence of dementia in our study (3.5%) is almost identical to estimate of dementia prevalence in community-dwelling older Australians (3.7%) suggesting that dementia is no worse in hospitalised injured fallers [44]. Our finding of greater dementia prevalence in the least disadvantaged group in two of the three years of interest is puzzling. In view of the short time frame between variations in dementia prevalence, this finding appears to suggest data quality issues rather than true differences between SES groups in the prevalence of dementia. The prevalence of renal disease in our study population (4.8%) is very similar to that reported for Victorian patients 18+ years (4.9%) [25].

Our finding that men hospitalised due to a fall had a higher comorbidity burden than women is supported by the literature [36,42]; however, this difference does not appear to have an impact on the average cumulative LOS per patient for 2005-6 to 2007-8. On the other hand, differences between the sexes in the rank order of comorbidities beyond the top three suggest that when prioritising modifiable risk factors for falls prevention programs gender differences should be taken into account.

On the basis of age, gender distribution and main injury types, our study population appears to be representative of falls hospitalisation cases generally, and hence our results might have wide generalisability. The cluster analysis produced three clusters--cancer, stroke and its sequelae, and neurological diseases--which are reasonably robust regardless of the choice of similarity measure. The separation of cancer into its own cluster appears to be reasonable because cancer is a disparate group of diseases. Although the most common risk factors for cancer, viz. age, smoking, poor diet, obesity and physical inactivity, are shared by many other comorbidities of interest [45], risk factors for a specific cancer may be unique. The partition of stroke into its own cluster seems to be inconsistent with current knowledge about common risk factors between stroke, diabetes and cardiovascular disease [45]; however, this cluster may simply reflect the fact that paraplegia is a sequelae of stroke and in community-dwelling fallers this comorbid pair is an independent problem. The neurological diseases cluster suggests that delirium and dementia are closely related, and this finding is supported by other epidemiological and biological research [46,47]. Since dementia is secondary to Parkinson's disease [48], the addition of Parkinson's disease to the dementia/delirium subcluster to form the neurological diseases cluster is consistent with current knowledge.

Two clusters (J1 and J3 in Figure 2 and Y1 and Y2 in Figure 3) are somewhat changeable depending on which similarity measure was chosen. This finding can be explained by differences between the Jaccard and Yule's Q coefficients in how joint absences were treated (see Figure 1) [29]. Given that 14 comorbidities were included in the cluster analysis, it would seem reasonable to treat joint absences as non-informative and exclude them from consideration as was the case with the Jaccard coefficient. The literature, in particular research on the biological relationships between diabetes, cardiovascular disease and renal disease, supports the clustering of comorbidities in J1 and J3 more than Y1 and Y2 [49]. The inclusion of osteoporosis in the J1 cluster is also consistent with recent biological and epidemiological evidence that links this condition with cardiovascular disease [50].

Our findings on the impact of comorbidity on length of hospital stay and the interrelationships between comorbidities have important implications for falls prevention strategies. Since patients with comorbidity stay in hospital longer and hence consume more hospital resources than those without comorbidity a targeted strategy focusing on the former using proven fall prevention interventions may be more cost-effective than a generalised strategy involving all community-dwelling older persons. Further research would be required before this approach may be adopted, in particular (a) studies assessing the impact of comorbidity or multicomorbidity on the cost-effectiveness of proven falls interventions; (b) studies investigating the need (or otherwise) for more complex falls interventions for older people with a particular comorbidity or combination of comorbidities; (c) studies exploring the feasibility and complexity of integrating falls prevention interventions into currently available intervention programs for individual comorbidities; and (d) studies examining options for prioritising falls prevention programs based on comorbidity status. Given that more than one-half of patients with multicomorbidity had either diabetes or renal disease, and in view of the interrelationships between these two comorbidities with each other and with the cardiopulmonary comorbidities in the cluster J1, a case could be made for people with diabetes and renal disease to be considered as a starting point for targeted falls prevention, provided that interventions effective in the general community-dwelling older people are as effective in patients with diabetes and/or renal disease.

The comorbidity clusters identified by our study provide the basis for further research into the causes and consequences of comorbidity clustering in terms of falls and/or injury outcomes, functional disability and survival. The elucidated interrelationships between comorbidities suggest that fallers with multicomorbidity of chronic diseases might have complex medical needs and hence an integration of follow-up services/programs for them within currently available models of chronic disease management might be desirable [51]. This might have a flow-on effect on the development and provision of clinical practice guidelines and clinical teaching for the management of hospitalised falls patients.

## Limitations

The category of patients in the VAED used to represent community-dwelling people includes people from prisons, armed forces base camps/hospitals, supported residential facilities (excluding nursing homes) and special accommodation houses [17]. Some of the patients included in our datasets would have resided in one of these facilities prior to hospitalisation; however, the VAED does not contain supplemental information to complement descriptions of accommodation categories. Finally, the lookback study was not comprehensive because we only had access to patients' previous hospitalisation records if these were fall-related.

## Conclusion

Comorbidity is common among community-dwelling older people hospitalised for fall-related injuries and is associated with a significant increase in the average cumulative LOS per patient. This study has identified five distinct, biologically plausible clusters of comorbidity, the interrelationships between these clusters and the intra-relationship between comorbidities within each cluster. These findings provide the basis for further research into the consequences of comorbidity clustering in terms of falls and/or injury outcomes, and have particular relevance for falls prevention strategies, clinical practice and planning of follow-up services for fallers.

## Competing interests

The authors declare that they have no competing interests.

## Authors' contributions

TV planned the study, applied for ethics approval, obtained hospitalisation data, defined and performed the statistical analysis, and drafted the manuscript and coordinated the contribution from CF and LD. CF and LD contributed to the conception, interpretation of data and critical appraisal of the manuscript. CF also contributed to aspects of the analytic approach. All authors read and approved the final manuscript.

## Pre-publication history

The pre-publication history for this paper can be accessed here:

http://www.biomedcentral.com/1471-2318/11/45/prepub
